# Editorial for Special Issue “Molecular Mechanisms and Signaling Pathways in Melanoma”

**DOI:** 10.3390/cancers15194675

**Published:** 2023-09-22

**Authors:** Alessio Giubellino, Carlos Torres-Cabala

**Affiliations:** 1 Department of Laboratory Medicine and Pathology, University of Minnesota, Minneapolis, MN 55455, USA; 2 Masonic Cancer Center, University of Minnesota, Minneapolis, MN 55455, USA; 3 Department of Pathology, The University of Texas MD Anderson Cancer Center, Houston, TX 77030, USA; ctcabala@mdanderson.org

Melanoma represents the leading cause of death from cutaneous malignancy [1], despite the development and deployment of novel targeted therapies (BRAF and MEK inhibitors) and immunotherapy [2]. Indeed, these treatment regimens are often short-lived and, despite being initially effective, are frequently followed by relapse.

Melanomagenesis and melanoma tumor progression are multistep processes, involving several cellular mechanisms and intracellular signaling pathways. One of the leading signaling pathways involved in melanoma is the MAPK signaling pathway, as exemplified by the leading driver mutations in the *BRAF*, *NRAS*, and *NF1* genes, as well as the involvement of receptor tyrosine kinases, such as KIT [3]. Thus, the exploration of these and other molecular mechanisms may help in uncovering new drug targets or potential drug combinations to be tested in clinical trials, with the aim of achieving FDA approval for routine treatment.

For this Cancers Special Issue, we collected relevant review articles and original research articles that summarize our current knowledge and advance our understanding of the pathogenesis of, and target therapies for, melanoma.

Among the receptor tyrosine kinase family, MET, the receptor for hepatocyte growth factor (HGF), has emerged as a potential key player in melanoma [4]. In their article, Song et al. [5] highlight how MET inhibition with selective kinase inhibitors appears to influence the expression of the checkpoint protein PD-L1 through interference in the JAK-STAT signaling pathway. Moreover, a physical integration between MET and PD-L1 has been demonstrated, both via co-immunoprecipitation experiments and through co-localization immunofluorescence studies. This interplay offers the theoretical opportunity to target these proteins with a combination cocktail that utilizes selective inhibitors of these surface receptors.

As mentioned above, *NRAS* represents one of the main molecular drivers in melanoma but, unfortunately, no FDA-approved drugs directly targeting *NRAS* mutations are currently available. Banik at al. [6] have now demonstrated that p38 has a selective tumor-suppression capability in NRAS-mutated melanoma, as it alters the actin cytoskeleton and autophagy-related targets. These findings open up opportunities to indirectly target the NRAS pathway with drugs modulating these proteins.

By using an RNA-sequencing strategy in conjunction with ChIP-seq techniques on melanoma cell lines with a high expression of GLI1-3 proteins in the hedgehog signaling pathway, Kurtovic et al. [7] were able to validate 21 targets via subsequent quantitative PCR. These proteins may represent potential targets for therapy, although they require further validation.

Two reviews within this Special Issue look into different and less-explored aspects of melanoma. Ye et al. [8] discuss the role of exosomal miRNAs in melanoma progression, by looking at their influence on metastasis, immune escape, and the tumor microenvironment. While this is an unconventional route to take in targeting melanoma, it may prove to be a viable alternative to current therapeutic regimens.

Finally, Pagliuca et al. [9] detail recent findings on melanoma tumor cell plasticity and phenotypic switching, noting that these important features are now recognized as hallmarks of cancer. Tumor plasticity appear to be regulated at the epigenetic level, and a debate continues on whether this feature is acquired through a selection process during tumorigenesis or pre-exists within the tumor in the early stages. Regardless, tumor plasticity represents another unconventional target that future research will, hopefully, allow us to include in our therapeutic toolbox.

Overall, novel therapeutic strategies are on the horizon for melanoma, and drug combinations appear to represent the most logical and rational approach to helping patients overcome ineffective treatments or treatment resistance, to ultimately improve survival and quality of life.

## References

[1] Siegel R.L., Miller K.D., Wagle N.S., Jemal A. (2023). Cancer statistics, 2023. CA Cancer J. Clin..

[2] Jenkins R.W., Fisher D.E. (2020). Treatment of Advanced Melanoma in 2020 and Beyond. J. Investig. Dermatol..

[3] Schadendorf D., Fisher D.E., Garbe C., Gershenwald J.E., Grob J.-J., Halpern A., Herlyn M., Marchetti M.A., McArthur G., Ribas A. (2015). Melanoma. Nat. Rev. Dis. Primers.

[4] Zhou Y., Song K.Y., Giubellino A. (2019). The Role of MET in Melanoma and Melanocytic Lesions. Am. J. Pathol..

[5] Song K.Y., Han Y.H., Roehrich H., Brown M.E., Torres-Cabala C., Giubellino A. (2023). MET Receptor Tyrosine Kinase Inhibition Reduces Interferon-Gamma (IFN-γ)-Stimulated PD-L1 Expression through the STAT3 Pathway in Melanoma Cells. Cancers.

[6] Banik I., Ghosh A., Beebe E., Burja B., Bertoncelj M.F., Dooley C.M., Markkanen E., Dummer R., Busch-Nentwich E.M., Levesque M.P. (2023). P38 Mediates Tumor Suppression through Reduced Autophagy and Actin Cytoskeleton Changes in NRAS-Mutant Melanoma. Cancers.

[7] Kurtović M., Piteša N., Bartoniček N., Ozretić P., Musani V., Čonkaš J., Petrić T., King C., Sabol M. (2022). RNA-seq and ChIP-seq Identification of Unique and Overlapping Targets of GLI Transcription Factors in Melanoma Cell Lines. Cancers.

[8] Ye Q., Li Z., Li Y., Li Y., Zhang Y., Gui R., Cui Y., Zhang Q., Qian L., Xiong Y. (2022). Exosome-Derived microRNA: Implications in Melanoma Progression, Diagnosis and Treatment. Cancers.

[9] Pagliuca C., Di Leo L., De Zio D. (2022). New Insights into the Phenotype Switching of Melanoma. Cancers.

